# Recent Advances on DNAzyme-Based Biosensors for Detection of Uranyl

**DOI:** 10.3389/fchem.2022.882250

**Published:** 2022-04-27

**Authors:** Yunlong Bai, Lechang Xu, Huining Chai, Lei Zhou, Guoping Jiang, Guangyao Zhang

**Affiliations:** ^1^ Beijing Research Institute of Chemical Engineering and Metallurgy, China National Nuclear Corporation, Beijing, China; ^2^ School of Environmental and Municipal Engineering, Qingdao University of Technology, Qingdao, China; ^3^ Intelligent Wearable Engineering Research Center of Qingdao, Research Center for Intelligent and Wearable Technology, College of Textiles and Clothing, State Key Laboratory of Bio-Fibers and Eco-Textiles, Qingdao University, Qingdao, China

**Keywords:** uranyl sensor, DNAzyme, environmental monitoring, detection, spectrum, electrochemistry

## Abstract

Nuclear facilities are widely used in fields such as national defense, industry, scientific research, and medicine, which play a huge role in military and civilian use. However, in the process of widespread application of nuclear technology, uranium and its compounds with high carcinogenic and biologically toxic cause a lot of environmental problems, such as pollutions of water, atmosphere, soil, or ecosystem. Bioensors with sensitivity and specificity for the detection of uranium are highly demand. Nucleic acid enzymes (DNAzyme) with merits of high sensitivity and selectivity for targets as excellent molecular recognition elements are commonly used for uranium sensor development. In this perspective review, we summarize DNAzyme-based biosensors for the quantitative detection of uranyl ions by integrating with diverse signal outputting strategies, such as fluorescent, colorimetry, surface-enhanced Raman scattering, and electrochemistry. Different design methods, limit of detection, and practical applications are fully discussed. Finally, the challenges, potential solutions, and future prospects of such DNAzyme-based sensors are also presented.

## Introduction

Uranium, a radioactive metal element, is a significant raw material for the nuclear industry including nuclear power, nuclear weapons, scientific research, and nuclear medicines. Uranium-based nuclear energy effectively reduces global environmental problems such as global warming and energy depletion caused by fossil energy. Economic, efficient, and clean nuclear energy has great development prospects. Whereas, with the development and application of nuclear technology, the radioactive wastes containing uranium will gradually infiltrate environmental media, such as water, soil, and atmosphere, and eventually enter the biosphere system, which will cause great damage to humans and ecosystems. Uranium with strong chemical toxicity and radiotoxicity will cause lasting disturbances and damage to the immune, reproduction, and hematopoietic systems of organisms (Domingo, 2001). The World Health Organization lists uranium as a priority environmental pollutant, stipulating that the concentration limit of uranium in drinking water is 30 ug/L (Ansoborlo et al., 2015). Uranium possesses a lot of forms under different conditions and the uranyl ion (UO_2_^2+^) is the most stable chemical form in aqueous solution. Many techniques have been developed for UO_2_^2+^ detection, including X-ray fluorescence spectroscopy, atomic emission spectrometry, inductively coupled plasma mass spectrometry, and high-performance liquid chromatography (Jamali et al., 2006; Jaison et al., 2011; Boulyga et al., 2016; Sanyal et al., 2017). These methods with high selectivity and sensitivity are high cost, requiring sophisticated instrument and tedious pre-treating procedures, which are difficult to achieve the goal of real-time and onsite detection. Therefore, bioensors with high sensitivity and specificity for the detection of uranyl ion have become increasingly necessary.

Nucleic acid enzymes, also called DNAzymes, are isolated through *in vitro* selection (Liu et al., 2009). DNAzyme typically is composed of a substrate strand and an enzyme strand, and the two DNA strands are partially complementary hybridized by base pairing to form a double-stranded system. The presence of target ion activates the activity of DNAzyme and the ribo-adenosine (rA) on the substrate chain is cleavaged. The released target ion subsequently interacts with another DNAzyme, resulting in a signal-amplifying effect. DNAzymes with high metal-binding affinity and specificity show great promise as molecular tools in the design of diverse biosensors and nanodevices, benefiting from their unique characters, including low nonspecific adsorption, good stability, and easy preparation (Zhou et al., 2017; Jouha and Xiong, 2021). Moreover, the recycling of target molecule properties makes DNAzymes outstanding signal amplifiers for enzyme-free and highly sensitive detection of many different metal ions (Saidur et al., 2017; Jouha and Xiong, 2021; Khan et al., 2021). UO_2_^2+^-specific DNAzyme was firstly selected by Lu group and a fluorescent sensor was developed simultaneously (Liu et al., 2007). In the past few years, quite a few amplified sensing platforms had been established for the quantitative detection of UO_2_^2+^ (Farzin et al., 2019; He and Hua, 2019; Wu et al., 2019; Wang et al., 2022). In this review, we only focused on the application of various types of DNAzymes-based methods with diverse signals outputting approaches to achieve quantitative detection of uranyl ion. Design strategies, detection limits, and detection ranges were comprehensively compared. Such DNAzymes based sensors are expected to show great potential in environmental monitoring and nuclear emergency.

## Applications of Different Types of Dnazyme-Based Sensors

DNAzymes as the recognition element are suitable for the fabrication of uranyl sensors. Target molecules induced huge changes in structure and conformation of DNAzymes to produce diverse signal outputting including fluorescence, electrochemistry, colorimetry, and surface-enhanced Raman scattering (SERS). Table 1 summarized the DNAzyme-based biosensors for the detection of UO_2_^2+^ by integrating with different signal output types including fluorescent, electrochemistry, colorimetry, and SERS.

**TABLE 1:** DNAzyme-based biosensors for the detection of uranyl ion.

Sensor Type	Design Method	Detection Limit	Dynamic Range	Ref.
Fluorescence	DNAzyme-FAM-Quencher	45 pM	1–400 nM	Liu et al. (2007)
Fluorescence	DNAzyme-Cy3-BHQ	45 pM	45 pM–20 µM	Wu et al. (2013)
Fluorescence	DNAzyme-AuNPs-fluorophore	25 pM	0.1–60 nM	Xiong et al. (2020)
Fluorescence	DNAzyme-DNA-SG	0.06 ng/ml	0.2–200 ng/ml	Zhu et al. (2019)
Fluorescence	DNAzyme-FAM-AuNPs	13 pM	30 pM–5 nM	Yun et al. (2019)
Fluorescence	DNAzyme-HCA-AuNPs-fluorophore	0.1 pM	0.2–1,000 pM	Yun et al. (2018)
Fluorescence	DNAzyme-2-aminopurine	9.6 nM	5–400 nM	Wang et al. (2019)
Fluorescence	DNAzyme-C3 Spacer	0.19 nM	2–1,000 nM	Feng et al. (2019)
Fluorescence	DNAzyme-FAM-MoS_2_	2.14 nM	5–100 nM	Zhang et al. (2015)
Fluorescence	DNAzyme-GO-NMM	86 pM	0.29–30 nM	Li et al. (2015)
Fluorescence	FAM-DNAzyme-DABCYL	0.6 nM	1–60 nM	Zhou et al. (2016)
Fluorescence	DNAzyme-SG-NMM	11.47 nM	10–1,000 nM	Yang et al. (2021)
Colorimetry	DNAzyme-SG-TMB-H_2_O_2_	0.08 μg/L	0.5–500 μg/L	Huang et al. (2018)
Colorimetry	MBs-DNAzyme-HCR-TMB-H_2_O_2_-HRP	0.33 nM	0.14–4.1 nM	Zhang et al. (2017)
Colorimetry	MBs-DNAzyme-RCA-TMB-H_2_O_2_	37 pM	74 pM–37 nM	Cheng et al. (2017)
Colorimetry	MBs-DNAzyme-AuNPs-TMB-H_2_O_2_	7 pM	74 pM–56 nM	Zhang et al. (2016)
Colorimetry	Hydrogel-DNAzyme-AuNPs	14 nM	50–800 nM	Huang et al. (2016)
Colorimetry	DNAzyme-AuNPs	4.09 μM	13.6–150 μM	Zhou et al. (2013)
Colorimetry	DNAzyme-litmus	15 μg/L	1.5–15 μg/L	Manochehry et al. (2018)
SERS	Rhodamine-DNAzyme-AuNPs	1.6 nM	2.5–100 nM	Jiang et al. (2013)
SERS	Cy5- DNAzyme-Au nanowire	1 pM	1 pM–100 nM	Gwak et al. (2016)
SERS	RhB -DNAzyme-ZnO-Ag	3.71 fM	0.1 pM–0.1 μM	He et al. (2019)
SERS	RhB -DNAzyme-ZnO-Ag	0.72 pM	1 pM–0.1 μM	He et al. (2020b)
SERS	DNAzyme -DNA Hydrogel- RhB	0.838 pM	1 pM–0.1 μM	He et al. (2020a)
Electrochemistry	DNAzyme-Ferrocene	1 nM	2–14 nM	Tang et al. (2013)
Electrochemistry	DNAzyme-AuNPs-Hexaammineruthenium (III)	5 pM	13 pM–0.15 nM	Ma et al. (2014)
Electrochemistry	DNAzyme-AuNPs-MB	8.1 pM	10–100 pM	Cao et al. (2020)
Electrochemistry	DNAzyme-HCR-MB	20 pM	0.05–4 nM	Yun et al. (2016b)
Electrochemistry	DNAzyme -CHA-MB	2 pM	10 pM–1 nM	Yun et al. (2016a)

BHQ: carboxylic acid; AuNPs: gold nanoparticles; SG: SYBR, green I; FAM: 6-carboxylfluorescein; HCA: hairpin catalytic assembly; MoS_2_: molybdenum disulfide; GO: graphene oxide; NMM: N-methyl-mesoporphyrin IX; MB: methylene blue; CHA: catalyzed hairpin assembly; HCR: hybridization chain reaction; TMB: 3,3′,5,5′-Tetramethylbenzidine; RCA: rolling circle amplification; SERS: Surface-enhanced Raman scattering, RhB: Rhodamine B.

### DNAzyme-Based Fluorescent Sensors for UO_2_^2+^ Detection

Fluorescence techniques are commonly used for the detection of diverse targets by measuring the change of fluorescence emission. DNAzyme as affinity ligand was applied in fluorescence sensors. Lu group reported a DNAzyme-based gold nanoparticles (AuNPs) sensor for uranyl ion detection (Wu et al., 2013). AuNPs were functioned with uranyl-specific DNAzyme and the substrate strand was modified with a fluorophore/quencher at 5′ and 3’-end respectively. In the absence of UO_2_^2+^, the fluorescence of fluorophore was quenched by both quencher and AuNPs. Upon uranyl ion binding, the cleavage of rA resulted in the release of fluorophore and the enhanced fluorescence enabled the sensitive detection of UO_2_^2+^ in living cells. Xiong et al. presented a DNA tweezer probe for the fluorescent detection of UO_2_^2+^. DNAzyme catalytic cleavage strategy was used for signal enhancement (Xiong et al., 2020). AuNPs and fluorophore were fixed at the ends of DNA tweezer. In the presence of target UO_2_^2+^, DNAzyme cleaved substrate linker DNA sequence, the enhanced fluorescent signal allowed the detection of target ions, and the limit of detection was 25 pM UO_2_^2+^.

**FIGURE 1:**
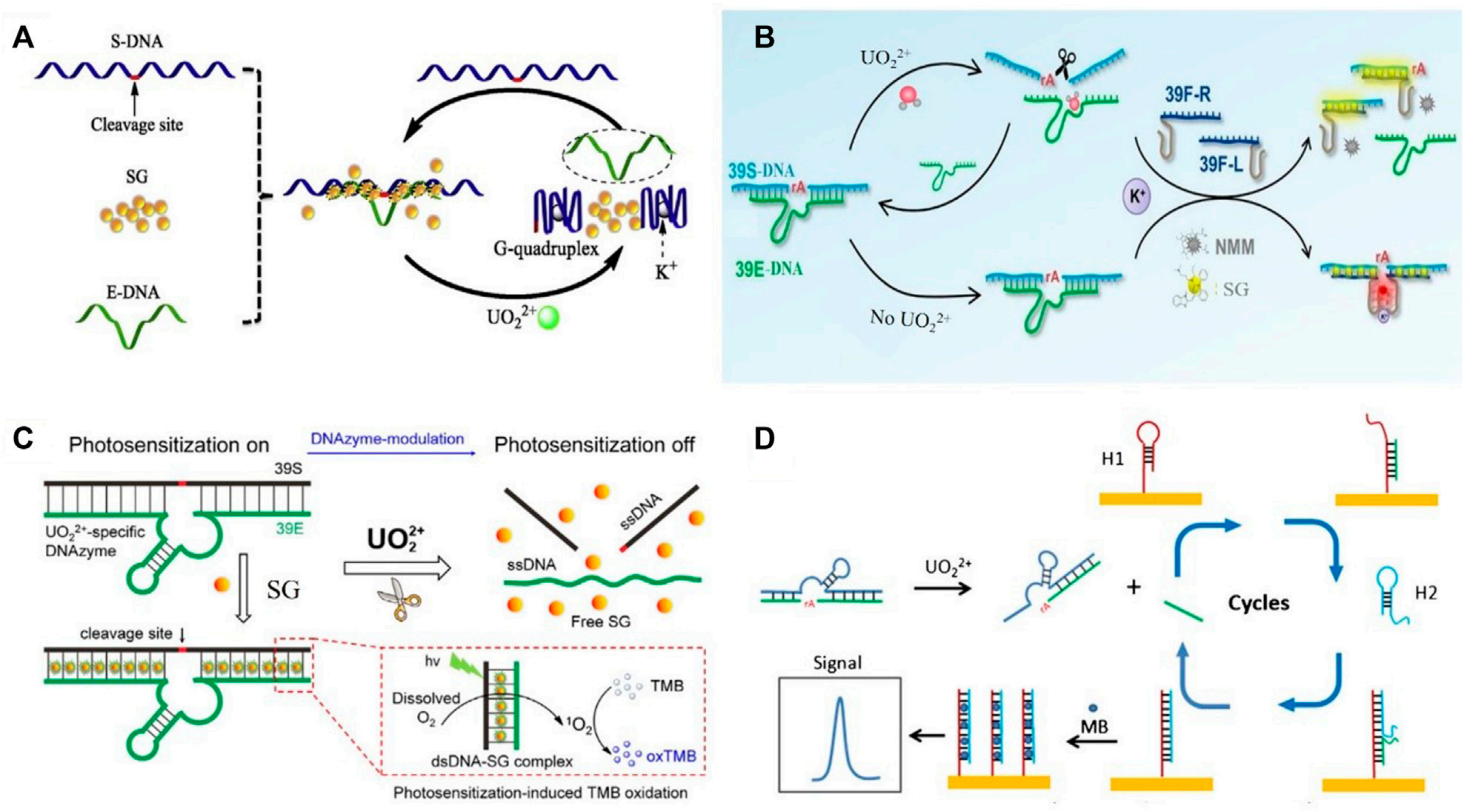
DNAzyme-based biosensors for the detection of uranyl ions by integrating with diverse signal outputting strategies in previous reports **(A)** G-quadruplex-assisted enzyme strand recycling-based fluorescent uranyl ion sensor. Reprinted with permission (Zhu et al., 2019), Copyright 2019 Elsevier B.V. **(B)** Ratiometric fluorescent biosensor for uranyl detection. Reprinted with permission (Yang et al., 2021). Copyright 2021 Elsevier B.V. **(C)** Electrochemical biosensor for uranyl detection based on DNAzyme and CHA. Reprinted with permission (Yun et al., 2016a). Copyright 2017, Royal Society of Chemistry **(D)** DNAzyme-modulated SG photosensitization colorimetric sensor for detection of UO_2_^2+^. Reprinted with permission (Huang et al., 2018). Copyright 2017, Royal Society of Chemistry.

Zhu et al. reported a G-quadruplex-assisted enzyme strand recycling strategy-based fluorescent sensor for detecting UO_2_^2+^ as shown in Figure 1A (Zhu et al., 2019). Such approach contained enzyme strand (E-DNA), cleaved substrate strand (S-DNA), and SYBR green I (SG). In the presence of target UO_2_^2+^, DNAzyme was activated and further cleaved the S-DNA containing G-quadruplex sequence at the both ends. The formation of G-quadruplex helped the separation between E-DNA and S-DNA, which obviously improved the recycle utilization of E-DNA. The fluorescent signal of SG, a DNA intercalating dye, positively correlated with the amount of target UO_2_^2+^. The limit of detection was 200 pM UO_2_^2+^.

A variety of signal amplification strategies were also applied to the design of UO_2_^2+^ sensor based on DNAzyme cleavage. Entropy-driven amplification and DNAzyme circular cleavage amplification-based fluorescent sensor was used for sensitive uranyl ion detection (Yun et al., 2019). The first amplification, entropy-driven amplification, was initiated by a DNA fragment coming from the cleavage of DNAzyme in the addition of target UO_2_^2+^. Two DNA sequences released from the entropy driven amplification were partly complementary, which was an E-DNA of Mg^2^-specific DNAzyme. The second amplification, DNAzyme circular cleavage amplification, subsequently was activated. The formation of E-DNA circularly cleaved S-DNA-FAM probes decorated AuNPs. The following recovery of fluorescent signal enabled the sensitive detection of UO_2_^2+^ and the limit of detection was as low as 13 pM. An enzyme-free dual amplification-based fluorescent sensor for ultra-sensitive detection of UO_2_^2+^ was reported by Huang et al. (Yun et al., 2018). The hairpin catalytic assembly (HCA) reaction and DNAzyme-strand recycling were used in such strategy. In the absence of UO_2_^2+^, dye-labeled hairpins absorbed on the surface of AuNPs and aggregation of AuNPs were prohibited. The addition of UO_2_^2+^ triggered HCA reaction between the three hairpins. The formed rigid DNA triangles with negatively charged released from the negatively charged AuNPs. Turn-on fluorescent signal achieved the sensitive detection of UO_2_^2+^ and the limit of detection was as low as 0.1 pM.

Wang et al. reported a fluorescent DNAzyme beacon probe for uranyl ion detection by embedding 2-aminopurine into the middle of S-DNA instead of labelling fluorescent dyes at the ends of S-DNA (Wang et al., 2019). 2-aminopurine, a fluorescent analog of adenosine, emitted fluorescence signals in a single-strand DNA (ssDNA); however, it was significantly quenched in the hybridized double-stranded DNA (dsDNA) due to the base-stacking interaction. In the presence of UO_2_^2+^, fluorescence signal recovered upon the cleavage of DNAzyme. Turn-on sensing process enabled the quantitative detection of UO_2_^2+^. The original catalytic activity of DNAzyme was hugely enhanced by inserting a C3 spacer. The length of flexible linkers and site of insertion were fully studied (Feng et al., 2019). Such modified DNAzyme was used in a fluorescent sensor and the detection limit was 0.19 nM UO_2_^2+^.

Nanosheets with merits of great adsorption of DNA probes and excellent fluorescence quenching effect were commonly used for the fabrication of fluorescence sensors. Fu group presented a fluorescent biosensor for the simple and rapid detection of UO_2_^2+^ in aqueous environment (Zhang et al., 2015). Such an approach used DNAzyme as target recognition element and molybdenum disulfide (MoS_2_) nanosheets as the fluorescence quencher. In the presence of UO_2_^2+^, the cleavage occurred and the released FAM-labeled ssDNA adsorbed on the surface of MoS_2_ nanosheets, resulting in an obvious decreased fluorescence signal. The limit of detection of this turn-on sensor was 2.14 nM. Graphene oxide (GO) was used as a quencher to lower background fluorescence for amplified detection of UO_2_^2+^ (Li et al., 2015). The presence of targets led to the cleavage of rA in DNAzyme, resulting in the formation of G-quadruplexes, which could interact with N-methyl-mesoporphyrin IX (NMM) to cause an enhanced fluorescence intensity. Free ssDNA and NMM were adsorbed by GO for background reduction. The limit of detection was as low as 86 pM.

DNAzyme nanostructures for UO_2_^2+^ detection in living cells were developed (Zhou et al., 2016). The fluorescently quenched nanoprobes were decorated by ssDNAs containing the metal ion-dependent enzymatic and substrate sequences. The self-assembly formation nanostructure could specifically recognize target ions to recover fluorescent emissions. Increased fluorescent signals enabled the quantitative detection of uranyl ions. Yang et al. reported a ratiometric fluorescent DNAzyme sensor for UO_2_^2+^ monitoring (Yang et al., 2021). The fluorescent biosensor contained DNAzyme probes (39E-DNA and 39S-DNA) and the split G-quadruplex probes (39F-R and 39F-L) as shown in Figure 1B. The presence of 39S-DNA leaded the proximity of 39F-R and 39F-L to form G-quadruplex. In the presence of target ions, DNAzyme-induced the cleavage of 39S-DNA splitted 39F-R and 39F-L. The decreased fluorescent of NMM was linear with uranyl ion concentration. Meanwhile, SG was used to monitor the hybridization of G-quadruplex probes and 39S-DNA. The ratiometric signal of NMM and SG enabled the robust detection of UO_2_^2+^.

### DNAzyme-Based Colorimetric Sensors

Colorimetric technique was used to determine the concentration of targets in solution by measuring the absorbance of a specific wavelength, which could be performed by UV-vis spectrophotometry (Priyadarshini and Pradhan, 2017). The sensitivity of colorimetric technique was lower than that of fluorescence; however, the color change was easily captured by naked eyes, making it a facile and convenient method.

DNAzyme-based colorimetric sensors had been developed for UO_2_^2+^ detection via DNAzyme modulated photosensitization as demonstrated in Figure 1C (Huang et al., 2018). The dsDNA structure allowed SG to be located in, which could activate the photosensitization of SG for TMB (3,3′,5,5′-tetramethylbenzidine) oxidation, and chromogenic reaction occurred subsequently. In the presence of target UO_2_^2+^, dsDNA structure of DNAzyme was cleaved and SG was released. Therefore, the color was weakened due to the reduced TMB oxidation from SG. This colorimetric sensor offered a detection limit of 0.08 μg/L (UV−vis detection) and 0.5 μg/L (naked eye).

Fu group reported magnetic beads (MBs) and HCR-based colorimetric biosensor for uranyl ion detection (Zhang et al., 2017). The addition of UO_2_^2+^ cleaved DNAzymes immobilized on MBs surface to release ssDNA. The released ssDNA on MBs surface triggered HCR to capture a large amount of horseradish peroxidase (HRP). Upon the addition of TMB-H_2_O_2_ solution, the HRP-DNA-MBs conjugates could catalyze the H_2_O_2_-mediated oxidation of TMB, a color change from colorless to blue in solution was observed. This provided a sensitive and selective sensing platform for the visual or colorimetric detection of UO_2_^2+^. The proposed biosensor has high sensitivity and strong anti-interference capability. In addition, the same group described a UO_2_^2+^ sensor in combination with rolling circle amplification (RCA) (Cheng et al., 2017). DNAzyme functionalized on MBs was selectively cleaved in the presence of UO_2_^2+^. The released DNA chains then triggered RCA, which increased the sensitivity of such biosensor. The detection limit was 37 pM. AuNPs-based enzymatic catalysis amplification was applied for UO_2_^2+^ sensor development (Zhang et al., 2016).

DNAzyme-functionalized MBs was used for UO_2_^2+^ recognition, released short ssDNA, then fixed HRP-functionalized AuNPs to the surface of MBs. H_2_O_2_-mediated oxidation of TMB occurred. The limit detection was 7 pM.

A smart hydrogel sensor was designed and synthesized for rapid, portable, sensitive detection of UO_2_^2+^ (Huang et al., 2016). DNA-grafted polyacrylamide chains were utilized to crosslink with DNAzyme to form the DNA hydrogel. Colorimetric analysis was achieved by encapsulating AuNPs in the DNAzyme-crosslinked hydrogel. The presence of UO_2_^2+^ in the sample activated the cleavage of substrate strand from the enzyme strand, thereby decreasing the density of crosslinkers and destabilizing the hydrogel, which then released the encapsulated AuNPs. The dispersion of AuNPs would lead to the change of absorbance. The decreased signal value enabled the quantification of uranyl ion. DNAzyme-functionalized AuNPs were used for UO_2_^2+^ detection (Zhou et al., 2013). The cleavage of the substrate strand of DNAzyme in the presence of targets resulted in releasing a shorter duplex, leading to the aggregation of AuNPs. The changed signal allowed the sensing of UO_2_^2+^. A litmus test-based assay for colorimetric uranyl biosensor was developed by Manochehry et al., The addition of target ions produced a pH-increasing enzyme, which was recognized by litmus paper. The changed signal was linear with uranyl ion concentration (Manochehry et al., 2018).

### DNAzyme-Based SERS Sensors

SERS technique with advantages of rapid detection speed, high-throughput screening, and high sensitivity had great potential in the high-speed and sensitive detection of diverse targets molecules (Pilot et al., 2019; Fan et al., 2020). Jiang et al. showed a label-free DNAzyme-based SERS method for sensing uranyl ion (Jiang et al., 2013). Such an approach used rhodamine as the Raman signal probe. The addition of UO_2_^2+^ induced the cleavage of DNAzyme and the released ssDNA was adsorbed on the surface of AuNPs to form a stable conjugate. Subsequently, the combination of rhodamine and AuNPs-ssDNA conjugate gave a strong SERS signal. Gwak et al. presented a DNAzyme-based plasmonic nanowire interstice sensor for uranyl ion detection (Gwak et al., 2016). The DNAzyme reacted with target UO_2_^2+^ and released Cy5 labeled strand. The plasmonic nanowire interstice sensor sensitively captured the released strands, giving a strong Raman signal and the detection limit was 1pM.

He et al. described a reusable SERS-based microfluidic biosensor for rapid detection of UO_2_^2+^(He et al., 2019). When target ions were added to the solution, 5′-rhodamine B (RhB)-labeled DNAzymes were cleavaged in the microfluidic chip, ZnO-Ag nanosheet arrays modified with S-DNA, which was sequence-complementary with the RhB-labeled E-DNA. The hybridization of S-DNA and E-DNA fixed RhB close to the surface of ZnO-Ag. The increased Raman signal enabled the sensitive detection of UO_2_^2+^. In addition, the same group developed a recyclable SERS-microfluidic biosensor for UO_2_^2+^ detection (He et al., 2020b). ZnO-Ag hybrids arrays were designed as the reaction substrates. In the absence of UO_2_^2+^, RhB-labeled dsDNA formed a rigid structure and weak Raman signal was detected. Addition of uranyl triggered DNAzyme-cleavage reaction. RhB was dropped down from the surface of SERS substrates, leading to the variation of Raman signals. The detection limit of uranyl was 0.72 pM. Flexible DNAzyme-based hydrogel SERS sensor for the detection of uranyl ions was also developed by Wang group (He et al., 2020a). The presence of UO_2_^2+^ ions triggered the activity of DNAzyme to cleave the substrate strand; subsequently, the DNA hydrogel structure was destroyed to release RhB, leading to a changed Raman signal. The detection of limit of such approach was 0.84 pM.

### DNAzyme-Based Electrochemical Sensors

Electrochemical biosensors had become increasingly popular due to its simplicity, portability, low cost, and high sensitivity (Wongkaew et al., 2019). DNAzymes were employed to achieve target recycling for signal amplification in electrochemical sensors. Tan et al. developed a DNAzyme-based electrochemical sensor for sensitive uranyl ion detection (Tang et al., 2013). A split uranyl-specific DNAzyme decorated with ferrocene unit was immobilized on the surface of a gold electrode. In the presence of uranyl, the cleavage of S-DNA induced the ferrocene release from the electrode. The measurement of changed electrochemical signal enabled the quantitative detection of UO_2_^2+^.

AuNPs with large surface areas had the ability to absorb large number of DNAzymes and electroactive indicator, which was suitable for electrochemical sensor fabrication. Ma et al. presented a DNAzymes- and AuNPs-based electrochemical biosensor for uranyl detection (Ma et al., 2014). The addition of UO_2_^2+^ induced the cleavage of DNAzymes and electroactive indicators were removed from the electrode subsequently. Differential current signals were used for sensing uranyl. Cao et al. designed an electrochemical biosensor for UO_2_^2+^ detection by the integration of DNAzyme and different DNA-modified AuNPs network structure (Cao et al., 2020). Such an approach effectively increased the amount of methylene blue (MB), a commonly used electrochemical indicator. The presence of UO_2_^2+^ triggered the cleavage of DNAzyme. MB and DNA-AuNPs then released from the gold electrode. The detection of reduced electrochemical response allowed the sensitive uranyl sensing with a low detection limit of 8.1 pM.

Different amplification strategies, such as hybridization chain reaction (HCR) and catalyzed hairpin assembly (CHA), were used for electrochemical biosensor development. Yun et al. reported an ultrasensitive DNAzyme-based electrochemical sensor for uranyl detection (Yun et al., 2016b). DNAzyme was selectively cleaved in the presence of UO_2_^2+^. The released DNA chains triggered HCR, which increased the sensitivity of monitoring. The detection limit was 20 pM uranyl. An ultrasensitive electrochemical biosensor for uranyl detection based on DNAzyme and CHA was presented by Yun et al. (Yun et al., 2016a). As shown in Figure 1D, DNA was selectively cleaved in the presence of UO_2_^2+^. The released fragment hybridized with hairpin probe 1 (H1) immobilized on the gold electrode. The unfolding of H1 subsequently induced the hybridization with hairpin 2 (H2). The DNA fragment spontaneously dissociated from the surface and then initiated the next hybridization cycle. MB was added to intercalate into the dsDNA. Double magnification strategy enabled the sensitive detection of UO_2_^2+^ and the detection limit was 2 pM.

## Conclusion and Outlooks

In this review, the recent progresses of sensors functioned with DNAzymes for uranyl ion detection were summarized including the design strategies, limit of detection, and dynamic range. Sensitive sensing for uranyl ion had been achieved with different signal outputting approaches including fluorescent, electrochemistry, colorimetry, and SERS. Despite great progress has already been achieved in this area, there are still many challenges to be overcome. Firstly, even though most DNAzyme-based uranyl sensors immune to the interference of commonly used metal ions, Th^4+^ can still affect the detection results. Hence, it is an important task in the future to improve the selectivity of such biosensor to tolerance the interfering ions. Secondly, in order to improve the limit of detection, complex construction strategies including using of functional nanomaterials and DNA recycle amplification (e.g., HCR, RCA and CHA) still need to be designed. Low-cost, portability, and onsite sensing platforms are of great promise in future uranyl sensor application. Thirdly, miniaturization and intellectualization are the trend of uranyl sensor in the future. Quantitative detection of uranium based on mobile phones is a very attractive direction (Chen et al., 2018), and chemical hybridization of carbon dots and CdTe QDs were used for the fabrication of ratiometric fluorescent probe. The addition of uranyl ions greatly quenched the red fluorescence of CdTe QDs, whereas the green fluorescence of carbon dots kept constant, leading to an obvious color change. A smartphone was used to analyze the content of uranyl on the basis of captured signals. Onsite and point-of-care detection of uranyl ions was achieved. Therefore, the sensitivity needed to be improved, which was mainly because the amplification strategy was not adopted.

Nowadays, the fabrication of high-quality DNAzyme-based sensors with merits of rapidness, low cost, sensitivity, and selectivity will become more active due to the widely used nuclear energy applications and the combined effects of its radioactive and biological toxicity during the uranium exploitation. Along with the great progress in the field of sensor design, we believe that DNAzyme-based sensors will play a vital role in various applications in environmental monitoring and nuclear environmental protection.
